# Estrogen–Estrogen Receptor α Signaling Facilitates Bilirubin Metabolism in Regenerating Liver Through Regulating Cytochrome P450 2A6 Expression

**DOI:** 10.1177/0963689717738258

**Published:** 2018-01-16

**Authors:** Ta-Lun Kao, Yao-Li Chen, Yu-Ping Kuan, Wei-Chun Chang, Yu-Chen Ho, Shuyuan Yeh, Long-Bin Jeng, Wen-Lung Ma

**Affiliations:** 1 Graduate Institution of Clinical Medical Science and Graduate Institute of Biomedical Sciences, China Medical University, Taichung, Taiwan; 2 Department of Trauma and Critical Care, Changhua Christian Hospital, Changhua, Taiwan; 3 Department of Surgery, Changhua Christian Hospital, Changhua, Taiwan; 4 Department of Obstetrics and Gynecology, Sex Hormone Research Center, Organ Transplantation Center, China Medical University Hospital, Taichung, Taiwan; 5 Department of Urology, University of Rochester Medical Center, Rochester, NY, USA; 6 Department of Nursing, Asia University, Taichung, Taiwan

**Keywords:** estrogen receptor α, bilirubin, cytochrome P450, CYP2A6

## Abstract

**Background:**

After living donor liver transplantation (LDLT), rising serum bilirubin levels commonly indicate insufficient numbers of hepatocytes are available to metabolize bilirubin into biliverdin. Recovery of bilirubin levels is an important marker of hepatocyte repopulation after LDLT. Cytochrome P450 (CYP) 2A6 in humans (or cyp2a4 in rodents) can function as “bilirubin oxidase.” Functional hepatocytes contain abundant CYP2A6, which is considered a marker for hepatocyte function recovery. The aim of our study was to determine the impact of estradiol/estrogen receptor signaling on bilirubin levels during liver function recovery.

**Methods:**

We conducted a hospital-based cohort study of bilirubin levels after LDLT surgery in both liver graft donors and recipients, performed a transcriptome comparison of wild-type versus estrogen receptor (ER)α knockout mice and a bioinformatics analysis of transcriptome changes in their regenerating liver after two-third partial hepatectomy (PHx), and assayed in vitro expression of cytochrome (CYP2A6) in human hepatic progenitor cells (HepRG) treated with 17β-estradiol (E2).

**Results:**

The latency of bilirubin level reduction was shorter in women than in men, suggesting that a female factor promotes bilirubin recovery after liver transplantation surgery. In the PHx mouse model, the expression of the *cyp2a4* gene was significantly lower in livers from the knockout ERα mice than in livers from their wild-type littermates; but the expression of other bilirubin metabolism–related genes were similar between these groups. Moreover, E2 or bilirubin treatments significantly promoted CYP2A6 expression in hepatocyte progenitor cells (HepRG cells). Sequence analysis revealed similar levels of aryl hydrocarbon receptor (AhR; bilirubin responsive nuclear receptor) and ESR1 binding to the promoter region of *CYP2A6*.

**Conclusions:**

This is the first report to demonstrate, on a molecular level, that E2/ERα signaling facilitates bilirubin metabolism in regenerating liver. Our findings contribute new knowledge to our understanding of why the latency of improved bilirubin metabolism and thereby liver function recovery is shorter in females than in males.

## Introduction

Bilirubin is an end catabolic product of heme. Heme oxygenase catalyzes the degradation of heme to water-soluble biliverdin. Biliverdin is then reduced by biliverdin reductase to water-insoluble bilirubin. Normally, most of the circulating bilirubin is unconjugated and bound to plasma albumin. Bilirubin is cleared and taken up by hepatocytes and conjugated to glucuronic acid by hepatic UDP-glucuronosyltransferase 1 (UGT1A1) to form “direct” bilirubin. Direct bilirubin is water-soluble so that it can be excreted in bile and urine. Impaired bilirubin conjugation by UGT1A1 can result in clinical jaundice, which is a sign of hepatic insufficiency.^1,2^

Bilirubin above a threshold level is cytotoxic but also acts as a potent antioxidant through a biliverdin–bilirubin cycling mechanism, possibly to protect cells against lipid peroxidation. It protects cells at intracellular concentrations ranging from 0.01 to 10 µM and kills cells above 20 µM. Therefore, strict regulation of its intracellular level is required.^3,4^

A variety of substances induce many of the cytochromes in humans including CYP1A, CYP2A, CYP2B, CYP2C, CYP2E1, and CYP3A.^5^ Cytochrome P450 2A6 is highly expressed in liver, where it metabolizes numerous xenobiotics and endogenous toxins.^6^ Increasing evidence has shown the interactions of bilirubin with cytochrome P450 (CYP) and UDP-glucuronosyltransferase (UGT), as well as demonstrated the induction of cytochromes by nuclear receptors including constitutive androstane receptor (CAR), retinoid X receptor α (RXRα), and hepatic nuclear factor (HNF).^7–10^ Treatment of cryopreserved human hepatocytes with 40 µg/mL bilirubin, which corresponds to the unconjugated bilirubin level in hyperbilirubinemia, results in elevated CYP2A6 expression, and the bilirubin-induced increase in CYP2A6 is concentration dependent.^11^ Also, the treatment of HepG2 cells with a bilirubin concentration of 50 µg/mL or more induces the reporter gene (−3475/+14) of UGT1A1.^2^ Bilirubin additionally causes nuclear translocation of CAR, activates CAR, and induces UGT1A1, an essential enzyme involved in bilirubin glucuronidation and its own elimination.^10,12^ It has been reported that the heterodimer CAR/RXRα binds to DR4-like elements in the human CYP2A6 gene and is responsible for CYP2A6 induction.^13^ Furthermore, HNF-4 was shown to activate hepatic transcription of the mouse cyp2a-4 gene and dose-dependently regulate human hepatic CYP2A6 gene expression.^14,15^

The influence of bilirubin on the messenger RNA (mRNA) expression of CYPs, UDP-glucuronosyltransferase (UGT), and nuclear receptors in human hepatocytes was investigated previously. As nuclear receptors, estrogen receptors (ERs), such as ERα and ERβ, have an unclear role in CYPs’ involvement in bilirubin clearance. It is well known that serum bilirubin levels rise immediately after 2/3 partial hepatectomy (PHx), then recover to normal levels 2 wks later in patients with normal liver function; however, the return to normal levels is prolonged in patients with chronic hepatitis or parenchymal diseases. A steadily rising serum bilirubin level is a particularly ominous sign of liver failure. And in both men and postmenopausal women, serum estradiol levels increase after PHx. The objectives of the present study were to determine the impact of estradiol/ER signaling on bilirubin levels during liver function recovery and to investigate the role of ER in bilirubin metabolism.

## Materials and Methods

### Patients’ Data

A retrospective chart review study (from July 2005 to October 2012) was conducted in the Department of Surgery and Critical Care Medicine of Changhua Christian Hospital (CCH) to evaluate all recipients of living donor liver transplantation. The clinical data validation was approved by the institutional review board (IRB #121221) of CCH. The inclusion criteria were (1) hyperbilirubinemia with total bilirubin higher than 1.2 mg/dL and (2) elevated conjugated or direct bilirubin level (>0.5 mg/dL). No patients were excluded, not even patients who died. All the patients were admitted to the intensive care unit. Once stabilized, they were transferred to a surgical ward. Table 1 shows the comparison of operative, donor, and recipient characteristics between male and female patients.

**Table 1.** Patient Demography of Liver Transplantation Surgery.

Variable	Female (n = 29)	Male, (n = 80)	*P* Value
Patient characteristics			
Age	54.9 (18-72)	54.3 (16-72)	0.79
DM	13 (44.8%)	38 (47.5%)	0.8065
Hypertension	6 (20.7%)	19 (23.8%)	0.7401
Chronic renal insufficiency	1 (3.4%)	4 (5.0%)	0.729
Preoperative bilirubin	5.17 ± 4.9	6.80 ± 3.1	0.1529
Diagnosis			
HBV	12 (41.4%)	48 (60.0%)	0.0843
HCV	15 (51.7%)	31 (38.8%)	0.2253
HCC	9 (31.0%)	39 (48.8%)	0.0997
Alcohol	1 (3.4%)	23 (28.8%)	0.0048
IHD stone	2 (6.9%)	0 (0%)	0.0178
Fulminate hepatitis	1 (3.4%)	3 (3.8%)	0.9203
Liver cirrhosis	28 (96.6%)	78 (97.5%)	0.7913
Hepatic encephalopathy	2 (6.9%)	11 (13.8%)	0.3297
EV breeding	1 (3.4%)	8 (10.0%)	0.2713
Hepatorenoal syndrome	1 (3.4%)	10 (5.0%)	0.1659
Operative characteristics			
Anhepatic phase (min)	60.6 ± 10.6	53.3 ± 3.9	0.4217
Cold ischemia (min)	132.8 ± 10.6	142.0 ± 6.3	0.4552
Warm ischemia (min)	1.9 ± 0.6	1.9 ± 0.4	0.9935
Postoperative variable			
Sepsis	8 (27.6%)	19 (23.8%)	0.6801
Shock	2 (6.9%)	4 (5.0%)	0.6985
Acute renal failure	3 (10.3%)	6 (7.5%)	0.6315
Diarrhea	0 (0%)	4 (5%)	0.2191
Malnutrition	0 (0%)	4 (5%)	0.2191
Acute respiratory failure	5 (17.2%)	11 (13.8%)	0.6468
Pleural effusion	13 (44.8%)	23 (28.8%)	0.1146
Mortality	3 (10.3%)	7 (8.8%)	0.8231

EV: esophageal varices; HBV: Hepatitis B Virus; HCV: Hepatitis C Virus; HCC: Hepatocellular Carcinoma; IHD: Intrahepatic bile duct.

### Reagents and Cell/Tissue Culture

HepaRG cells (Invitrogen, Carlsbad, CA, USA; catalogue no. HPRGC10) were maintained following the vendor’s and previously published instructions.^16^ Briefly, the cells were cultured in Dulbecco’s modified Eagle’s medium containing 1 mg/mL d-glucose and supplemented with 0.3 mg/mL l-glutamine and 10% fetal bovine serum ((Invitrogen, USA). All cells were maintained at 37 °C in a humidified incubator with 5% CO_2_. 17β-Estradiol (E2; Sigma-Aldrich, St Louis, MO, USA) was dissolved in ethanol (EtOH), and bilirubin (Sigma-Aldrich, USA) was dissolved in dimethyl sulfoxide (DMSO, USA).

### Real time polymerase chain reaction

The method of gene expression measurement by real time polymerase chain reaction (RT-PCR) was previously described.^17^ At the treatment end point, the medium was removed, and total RNA was extracted with TRIzol reagent (Life Technologies Corporation, Carlsbad, CA, USA). The concentration and purity of RNA were determined with a spectrophotometer (Thermo, ND-2000c, Waltham, MA, USA). Reverse transcription was performed using the TaKaRa RNA PCR kit Avian Myeloblastosis Virus (AMV) Version 3.0 (Takara Bio Co., Ltd., Shiga, Japan) according to manufacturer’s instructions. Total RNA (400 ng) was mixed with reaction buffer, 5 mM MgCl_2_, deoxyNucleotide triphosphate (dNTP) mixture (1 mM each), RNase inhibitor (1 U/µL), and random 6 mers (2.5 µM) in a final volume of 20 µL. The reaction mixture was incubated at 30 °C for 10 min followed by 42 °C for 30 min and then heated at 95 °C for 5 min to inactivate the enzyme. PCR was carried out using the SYBRgreen mix (Cambrex Bio Science Rockland, Inc., Rockland, ME, USA). The primers used were forward sequence 5′ TGGCTATGAAGTCCTCCAGC 3′ and reverse sequence 5′ CAGTTCACGTCAACCTCCAC 3′. The number of cycles was optimized to fall within a linear amplification range. To standardize the amount of sample, the calculated amount of the gene of interest was divided by the calculated amount of the constitutively expressed 18 S gene in the sample. These normalized amounts were then used to compare the relative amount of target mRNA between different samples.

### Generation of ERα-KO mice*^18^*

The ERα-knockout (KO) mice beta-Actin Cre-ERaKnockout (ActbCre-ERα^loxP/loxP^) used in our study were kindly provided by professor Shuyuan Yeh, University of Rochester, NY, USA. In principle, we crossed transgenic ERα^loxP/loxP^ mice with ACTB-Cre (β-actin promoter-driven Cre recombinase) transgenic mice to generate male ERα knockout (ERα^–/–^) mice. The control mice were ERα^loxP/loxP^ mice without ACTB-Cre. The genotypes of mice were identified using PCR analysis of DNA obtained from tail biopsies that were lysed in buffer with 0.5 mg/mL proteinase K (Sigma, P2308, USA) overnight. Wild-type (WT) versus ERα^–/–^ male mice (aged 2 to 4 months) were compared in the studies. All protocols related to animals and the study protocol were reviewed and approved by the animal care and use committee of the China Medical University, and all animals were treated in accordance with National Laboratory for Experimental Animals guidelines.

### Two-third Partial Hepatectomy Surgery*^19^*

Two-third PHx surgeries were performed using a large abdominal incision and 2 separate ligatures to remove the lobes as described for liver regeneration experiments.^19^ In brief, after anesthetizing approximately 10- to 12-week-old mice with isoflurane, their skin was disinfected (10% Povidone-iodine), incised, and gently pulled down with a saline-moistened cotton tip to reveal the median lobe. The falciform ligament or membrane was cut with a curved microsurgery scissors. The 3-0 silk thread was placed on the base of the left lateral and median lobes using microdissecting forceps. With a cotton tip, the left lateral or median lobe was rotated to its original position. Although holding the right end of the suture with the microdissecting forceps, a noose was made around the lobes. Close to the base of the lobe as possible, the noose was tied and knotted, and the tied lobe was cut just above the suture using microsurgery curved scissors. Finally, we closed the peritoneum using 3-0 sutures, and the skin using 4-0 sutures. The animal was placed on a heating pad for recovery. After surgery, the mice were housed in individual cages as usual, and then regenerated livers and blood were obtained after sacrificing the mice on the postsurgical 4th day.

### The complementary DNA Microarray for Transcriptome Study*^20^*

The extracted RNA was sent for microarray analysis to Agilent Technologies (Foster City, CA, USA). Briefly, 0.2 µg of total RNA was amplified using a Low Input Quick-Amp Labeling kit (Agilent Technologies, CA, USA) and labeled with Cy3 (CyDye, Agilent Technologies, CA, USA) during the in vitro transcription process. A total of 0.6 µg of Cy3-labeled complementary RNA (cRNA) was fragmented to an average size of about 50 to 100 nucleotides by incubation in fragmentation buffer at 60 °C for 30 min. Correspondingly, fragmented labeled cRNA was then pooled and hybridized to Agilent SurePrint G3 Mouse GE 8×60 K Microarrays (Agilent Technologies) at 65 °C for 17 h. After washing and drying the microarrays by nitrogen gun blowing, they were scanned with an Agilent microarray scanner (Agilent Technologies) at 535 nm for Cy3. Scanned images were analyzed using Feature Extraction 10.5.1.1 software (Agilent Technologies), and the signal and background intensity for each feature was quantified using image analysis and normalization software.

### Data Mining for Known ERα Binding Sites*^16,21^*

The global ER interaction network refers to a network of genes that directly or indirectly interact or are functionally associated with ER. Several bioinformatics databases were used for further analysis. The TFcheckpoint (http://www.tfcheckpoint.org/) data set is used to identify transcription factors. TRANSFAC (http://www.biobase-international.com/product/transcription-factor-binding-sites) provides data on eukaryotic transcription factors, consensus binding sequences (positional weight matrices), and regulated genes.

### Analysis and Statistics

The Student’s *t* test was used to identify significant differences, and the χ^2^ statistic was used to investigate whether distributions of categorical variables differ between male and female groups. A *P* value less than 0.05 was considered significant. All data are reported as the mean ± standard error of the mean.

## Results

### The Restoration of Serum Bilirubin Concentration to Normal Levels Was Faster in Females than Males

**Figure 1.**
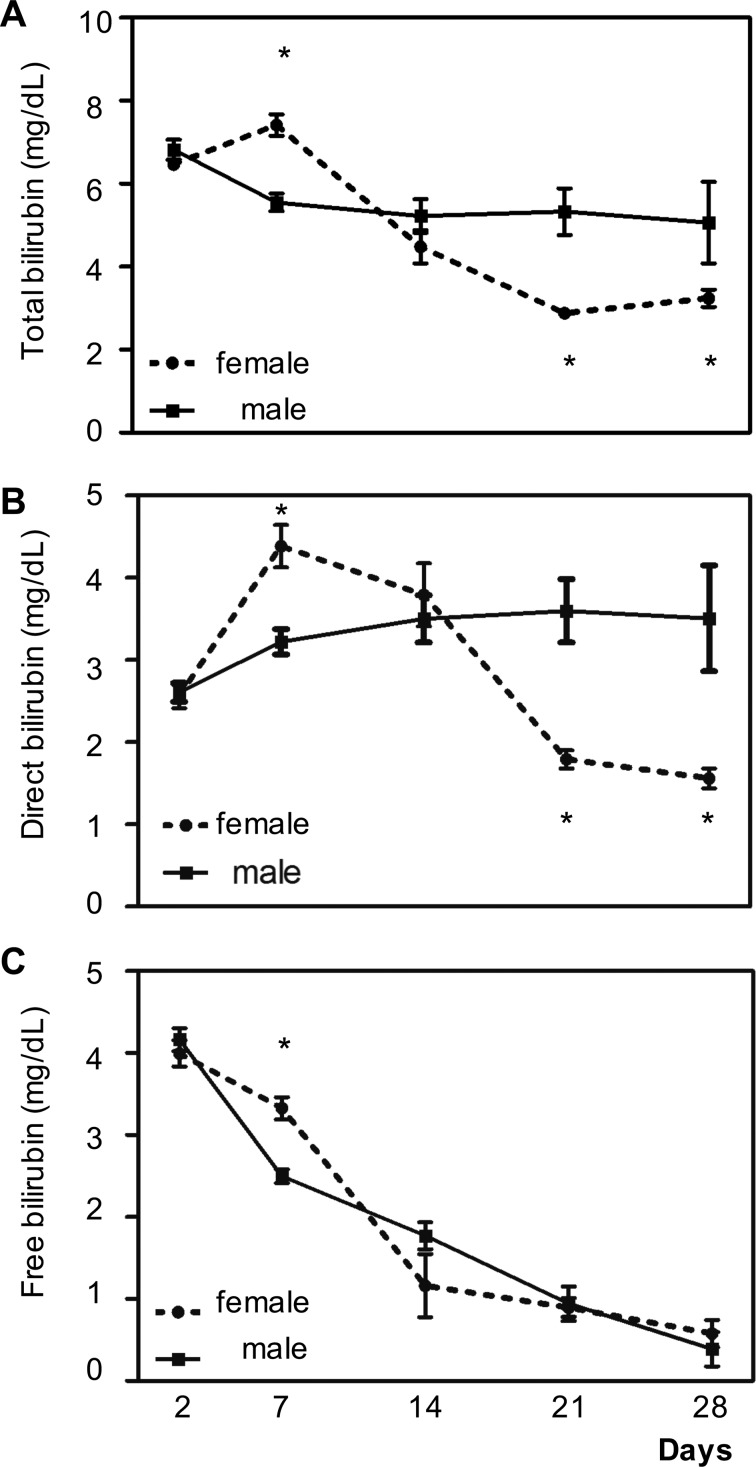
Gender difference in total and direct bilirubin level reductions measured at 2, 7, 14, 21, and 28 d after living donor liver transplantation surgery. Male patients = 80; female patients = 29. (A) Total bilirubin level. (B) Direct bilirubin level. (C) Free bilirubin level. *Indicates a *P* value less than 0.05, while **indicates a *P* value less than 0.001.

Serum bilirubin concentration reduction after LDLT surgery is considered to be a good outcome and an early predictor of good prognosis and functional liver recovery. To investigate how gender affects bilirubin level in the postoperative recovery, we retrospectively assessed patients’ serum bilirubin levels after LDLT in a single hospital cohort study. Liver function recovery mainly depends on pretransplant clinical disorders, donor liver quality, intraoperative variables, and perioperative clinical conditions. The major postliver transplant outcome determinant, injury due to prolonged cold and/or warm ischemia, has been associated with adverse postoperative outcomes including deteriorated liver function, increased bilirubin and aminotransferase levels, propensity for nosocomial infection, and sepsis often involving prolonged hospital stay. We can see no significant gender differences either in the underlying disease, etiology of liver failure, intraoperative characteristics, or postoperative clinical course. There are 2 forms of bilirubin in the human body: indirect (or unconjugated) bilirubin and direct (or conjugated) bilirubin. Total bilirubin is both combined. We measured total (Fig. 1A) bilirubin level and the levels of the direct (conjugated form; Fig. 1B), and unconjugated free (Fig. 1C) forms and found an initial increase in total serum concentration and the serum concentration of the direct form on postsurgical days 2 to 7, and then a decline at day 14, 21, and 28. The decline in total and direct bilirubin was faster in females. Interestingly, the level of free-form bilirubin was higher in females on day 7. This difference in the biphasic course bilirubin level recovery after LDLT suggests that female factors could be promoting post-LDLT recovery after day 7.

### ERα Transcriptome Analysis Revealed cyp2a4 Might Be Responsible for Reducing Bilirubin Levels in Regenerating Liver

**Figure 2.**
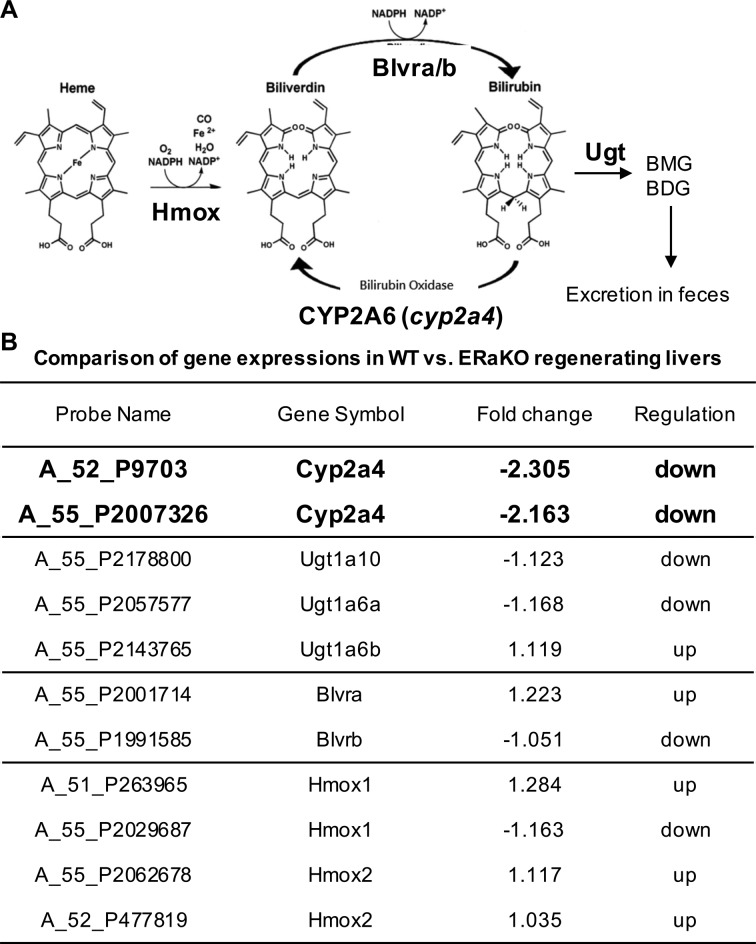
E2/ERα signals induce cyp2a4 expression during liver regeneration. (A) The diagram shows the metabolic pathway from heme to BMG/BDG (bilirubin mono/diglucuronide). The heme moiety can be oxidized by Hmox (heme oxygenase) to form biliverdin. Then biliverdin can be reduced by Blvr-a/b (biliverdin reductase-a and biliverdin reductase-b) to form bilirubin. This process can be reversed by the hepatic enzyme cyp2a4 in rodents (or CYP2A6 in humans). Bilirubin can further be catalyzed by Ugt (UDP-glucuronosyltransferase) to form BMG and BDG. Finally, BMG and BDG are excreted through feces. (B) The table compares the gene expressions of enzymes involved in hepatic bilirubin metabolism between total ERα knockout (ERα-KO; *n* = 3) mice and wild-type (WT; *n* = 3) mice. The genes (labeled in red) had significantly altered expressions (>2-fold; 2 probes to detect different gene regions on the complementary DNA [cDNA]). Others (labeled in black) had nonsignificantly altered expressions (less than 2-fold).

The metabolism of heme into bilirubin and biliverdin is illustrated in Fig. 2A. The heme moiety is oxidized by heme oxygenase (Hmox) to biliverdin, which is then reduced by biliverdin reductase (Blvr-a or Blvr-b) to bilirubin. The reduction is reversed by CYP1A6 (or cyp2a4 in rodents). Finally, the bilirubin can be converted by UGT to bilirubin monoglucuronide (BMG) or bilirubin diglucuronide (BDG) and finally excreted in the feces.

To dissect estrogen regulation of bilirubin metabolism, we compared the transcriptomes of livers from WT and ERα knockout mice that had undergone two-thirds PHx surgery. First, we found the estradiol level is comparable in 2 genotypes of day 2 post-PHx surgery mice (WT = 136.98 ± 91.80 pg/mL; ERα-KO = 61.18 ± 37.35). Analyzing the acquired transcriptome data bioinformatically by gene set enrichment analysis of gene ontology terms, we found that cyp2a4 level was significantly decreased in ERα knockout mice (Fig. 2B). However, the levels of the other metabolic enzymes were comparable between WT and ERα knockout mice. These data indicate ERα might upregulate cyp2a4 expression to reduce bilirubin level in the regenerating liver.

### Both Estrogen and Bilirubin Levels Upregulate CYP2A6 in Vitro

**Figure 3.**
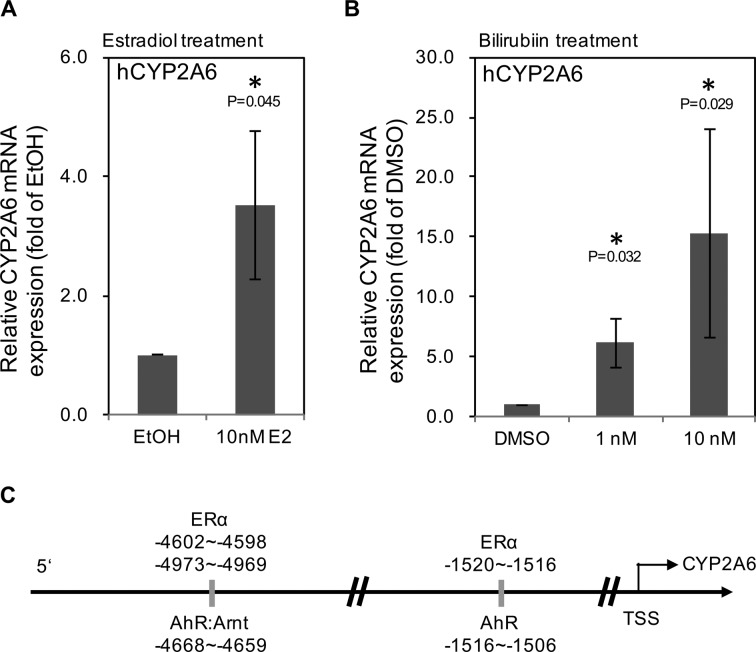
Estradiol/estrogen receptor (E2/ERα) and bilirubin-aryl hydrocarbon receptor (AhR) signals upregulate CYP2A6. (A) The CYP2A6 messenger RNA (mRNA) expression in human hepatic progenitor cells (HepRG) cells was enhanced by 10 nM E2 for 48 h. (B) The CYP2A6 mRNA expression in human HepRG cells was enhanced by 1 and 10 nM bilirubin for 48 h. (C) Analysis of the genome of the *cyp2a6* 5′-promoter region (0∼6000 bp from the transcription start site [TSS]). The putative ERα-binding regions (−1520 to −1516; −4602 to −4598; and −4973 to −4969) colocalized with AhR-binding regions (−1516 to 1506; and −4668 to −4659). All the gene expression values were from at least 3 experiments. *Indicates *P* value less than 0.05.

Since we found ERα could promote cyp2a4 expression in regenerating liver of mice, we suspected that the faster decrease in bilirubin level in females could be attributed to an estrogen effect on CYP1A6 expression. Therefore, human hepatocyte progenitor cells, HepRG, were treated with 10 μM E2 (similar to E2 levels in mice with regenerating liver) and evaluated for CYP2A6 mRNA expression. As shown in Fig. 3A, CYP2A6 expression was significantly upregulated by 10 μM E2 treatment. In addition, since bilirubin promotes CYP2A6 expression, we tested whether bilirubin (1 and 10 nM) could promote HepRG CYP2A6 expression in our system. Figure 3B shows that bilirubin upregulated CYP2A6 mRNA expression in a dose-dependent manner. In order to confirm that the interaction of E2/ERα signals with bilirubin regulates CYP2A6 expression, we analyzed the CYP2A6 promoter region and found that the ERα response elements were highly associated with the aryl hydrocarbon receptor (AhR) binding site in the CYP2A6 promoter region (Fig. 3C). Two of the 3 ERα binding sites (−1520, −4602, and −4973) were in the AhR binding region (which extends from −1516 to −4668). Considering the free bilirubin level was higher in females than males at day 7 (Fig. 1), we suggest that bilirubin may act on CYP2A6 expression through AhR and E2/ERα signaling to reduce bilirubin levels.

Collectively, the in vivo and in vitro data support the notion that estrogen binds to ERα to promote the oxidation of bilirubin to biliverdin through upregulation of CYP2A6 in patients (or cyp2a4 in rodents).

## Discussion

**Figure 4.**
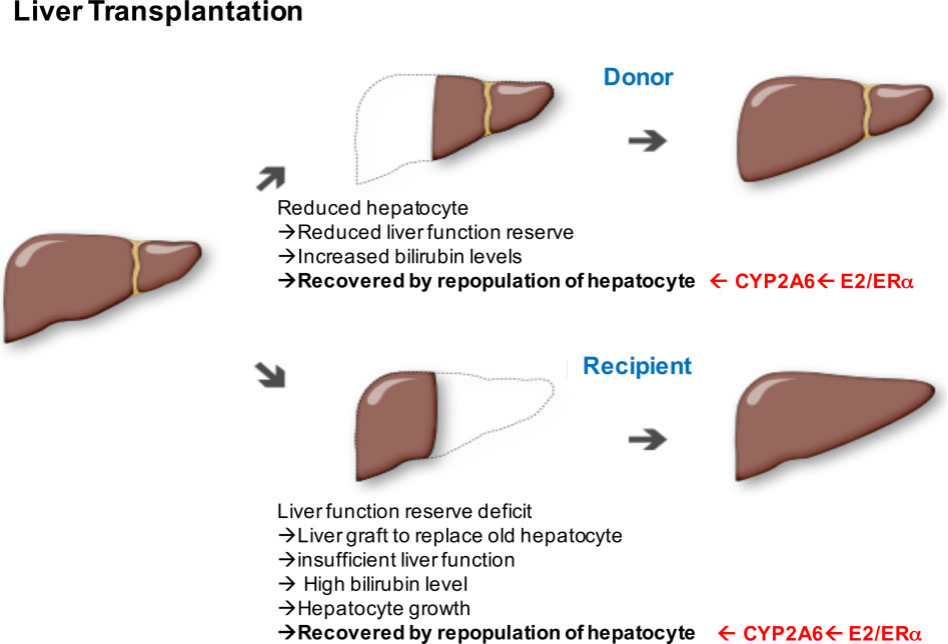
The diagram shows the cellular events involved in liver regeneration in donors and recipients of living donor liver transplantation (LDLT) transplants. The red-colored text indicates the roles played by the estradiol/estrogen receptor (E2/ERα)-cytochrome (CYP2A6) axis in bilirubin metabolism.

The clinical significance of early and prolonged postoperative hyperbilirubinemia after LDLT is a significant predictive factor of early liver graft failure and mortality.^22,23^ Postoperative hyperbilirubinemia in LDLT could be due to a variety of etiologies, including poor graft function, use of aged liver donors, small-for-size syndrome, inadequate liver graft preservation, surgical complications, acute cellular rejection, immunosuppressive drug toxicity, and so on.^23–35^ In the current study, the female sex affected bilirubin levels in the post-LDLT period. This finding provided the basis for proposing that estrogenic signals might promote liver function recovery. Interestingly, the knockout of ERα in the mouse did not change the level of the bilirubin metabolism enzyme UGT1A1 but significantly downregulated cyp2a4 level. This result suggests that ERα could be the important mediator of bilirubin level reduction during mouse liver regeneration and that CYP2A6 expression in humans might be the important mediator of the estrogenic signal in bilirubin level reduction. Indeed, CYP2A6 expression is significantly upregulated in human hepatic progenitor cells with E2. Besides, bilirubin itself was linked to ERα-induced promotion of CYP2A6 expression. The significance of this study is as follows and presented in Fig. 4.

### ERα Might Be the Promoter of Hepatocyte Repopulation in Both Donors and Recipients After LDLT

LDLT is a complex surgery and requires the regeneration of a partial liver graft, unlike deceased donor liver transplantation of the whole cadaveric liver, where regeneration has less of a role. Knowing more about partial graft regeneration will help improve treatment and thereby donor and recipient outcomes in LDLT.^36^ Liver regeneration (LR) capacity is critical for preserving liver function reserve after liver resection.^37^ There are 2 aspects of LR capacity: hepatocyte repopulation and hepatic progenitor differentiation to functional hepatocytes. Regarding the donor side of post-LDLT surgery (upper part of Fig. 4), after harvesting the liver graft, the remnant liver faces challenges associated with the diminished bulk of the liver. The loss of hepatocyte number causes deteriorated liver functions including transient elevation of liver enzymes, hyperbilirubinemia, hypoalbuminemia, and abnormal prothrombin time.^22^ The peak of aminotransferase elevation is reached its peak in first days after harvest, while that of serum bilirubin is reached on the third postoperative day.^38^ LR capacity increases due to insufficient liver function reserve. After a period of time, hepatocyte repopulation succeeds with the maturation of hepatocyte progenitors, recovery from hyperbilirubinemia, gradual return of liver function test values to normal within postoperative 30 d, and favorable survival rate.^38,39^ Thus, the E2/ERα-CYP2A6 axis discovered in this report plays a role in functional recovery after hepatocyte repopulation.

Regarding the recipient side of post-LDLT surgery (lower part of Fig. 4), the original deficient liver is replaced by a new functional liver but is still insufficient to restore the liver function to normal level. The insufficiency of hepatic function is reflected in the bilirubin level. In the recipients of right-lobe adult-to-adult living donor liver grafts, the liver regenerates rapidly and is almost complete after 2 wks as determined by abdominal magnetic resonance imaging scanning.^40^ Recipients with postoperative hyperbilirubinemia recover rapidly with good liver function. Therefore, the regeneration process is initiated via increasing hepatocyte numbers and maturation of hepatocyte progenitors. At this point, E2/ERα-CYP2A6 axis might play a role in hepatocyte maturation and recovery.

### Conclusion

Our study demonstrated that E2/ERα signaling increase in bilirubin metabolism might contribute to better post-LDLT surgery outcome and better hepatocyte function recovery during the liver regeneration process. Further analysis of E2/ERα’s role in LR on the molecular and systems levels is required.
